# (Cost)effectiveness of life review for Older Adults: Design of a randomized controlled trial

**DOI:** 10.1186/1471-2458-8-211

**Published:** 2008-06-13

**Authors:** Anne Margriet Pot, Anne-Sophie Melenhorst, Simone Onrust, Ernst T Bohlmeijer

**Affiliations:** 1 Netherlands Institute of Mental Health and Addiction (Trimbos-instituut), Utrecht, The Netherlands; 2 Department of Clinical Psychology, VU University, Amsterdam, The Netherlands; 3 Melenhorst Teksten, Heino, The Netherlands; 4 Department of Behavioral Sciences, University of Twente, The Netherlands

## Abstract

**Background:**

Depression in older adults is a serious health problem with a poor prognosis. There is a need for indicated preventive psychological interventions for older adults, that show to be promising in preventing depressive disorders.

**Methods/design:**

This manuscript describes the design of a study evaluating 'Looking for Meaning', a newly developed prevention course for older adults with depressive symptoms, based on life-review. Both clinical and economic effectiveness are evaluated in a pragmatic randomized controlled trial. The control condition of this 12-session preventive intervention is a 20-minute video movie. The primary outcome is symptoms of depression at post-treatment and follow-up (6 months after post-treatment). Secondary outcomes are symptoms of anxiety, satisfaction with life, mastery, reminiscence styles, quality of life, and health care costs. An additional result of this study is the insight into the working elements of the course, provided by the qualitative study. The qualitative data, mainly based on 20 open-ended interviews with participants, are to be analyzed with an emphasis on newly emerging insight.

**Discussion:**

This study will add to the existing scientific knowledge in several ways, especially by also including an economic evaluation and a qualitative study to gain insight into the working mechanisms of the course, both rather new in the field of life review. Positive results of this study will make an evidence-based intervention to improve public health among older people available.

**Trial registration:**

Current Controlled Trials Ltd, ISRCTN66645855

## Background

Depression in older adults is a serious health problem with a poor prognosis [1]. Major depression occurs in approximately 1,8% of the general population aged 55 and over, and minor depression in another 9,8% [2]. Considering the high prevalence rate of depressions in people aged 55 and older, there is a need for effective, low-threshold preventive interventions tailored to older adults.

Indicated prevention starts tackling disorders at the symptom level [3]. In contrast with universal prevention (general; aims at no specific target group) and selective prevention (focuses on risk groups with no symptoms yet), indicated prevention directly deals with the major risk factor for developing a full-blown depression particularly in old age: the presence of some of the depressive symptoms [4]. A recent meta-analysis shows that psychological interventions for older adults with depressive symptoms are indeed promising in preventing depressive disorders [5].

Several psychological interventions are currently available for reducing depression symptoms in older people, including psycho-educational approaches, cognitive behavioral therapy, psychodynamic therapy, and interpersonal therapy [6-8]. The use of life review is gaining popularity, because this type of intervention may pertain to older adults in particular. Meta-analyses have shown that life-review has significant and substantial effects on both depression and psychological well-being [9,10].

The first to emphasize the importance of life review in old age was Butler [11]. He observed that older adults increasingly review their lives as they grow older, and that they seem to do so naturally. He considered life review the expression of a universal mental process reflecting the need to come to terms with one's life when the end is approaching. Since Butler's observations, scientists and clinicians have shown a growing interest in life review, both as a phenomenon and as a method of treatment for older adults with depression [12-14].

Life review, can fulfill various functions for the individual; people review their lives for different reasons and with different motivations. An individual may apply more than one style. A particular reminiscence style is assumed to relate with more or less favorable outcomes in terms of mental health [15]. Integrative life review is most well-known, and implies an evaluation of one's past in order to accept negative events, resolve past conflicts, identify continuity of past and present, and, eventually, find meaning in life [16]. However, for some older adults life review can be depressing or burdensome. Especially when they tend to focus on negative past experiences, life-review may have a self-subversive rather than positive impact. Life review prompted by 'negative' motivations, such as boredom reduction or bitterness revival, are associated with undesirable clinical outcomes (e.g., anxiety, a low sense of self efficacy, dissatisfaction with life, or depressive symptoms). Transforming these styles and supporting 'positive' reminiscence may therefore promote older adults' mental health.

We developed an indicated prevention course, called 'Looking for Meaning', which is a well-structured type of life review with the underlying assumptions that reminiscence styles influence coping with depressive symptoms and can be changed or transformed by means of an intervention [17]. This course may prevent depressive symptoms from developing into a major depression. A pilot study with a previous version of this course exploring the impact of this approach showed promising results for the mental health of older adults [18]. A next step is to carry out a randomized controlled trial (RCT).

In this paper the design of the RCT on (cost)effectiveness of life review for older adults will be described. In order to be able to explicitly relate reminiscence styles to clinical outcomes, we also measured reminiscence styles and -functions. In addition, a qualitative study was designed to gain an insight into what participating in this course meant to the older adults and how life review in particular might have influenced coping with depression and other mental health issues in their lives. The specific research questions these studies aimed to answer were:

1. Does the prevention program Looking for Meaning lead to a significant reduction of depressive symptoms and a significant enhancement of wellbeing, satisfaction with life, and quality of life in elderly people with depressive symptoms, in comparison with a no-treatment control group?

2. Which prognostic factors (demographic, clinical symptoms, mastery), in combination with the intervention, predict lower or higher effects on depressive symptoms, wellbeing, satisfaction with life and quality of life?

3. What is the incremental cost-utility in the treatment group as compared to the control group?

4. Which are the working components of the intervention, leading to reduction of depression and enhancement of wellbeing, mastery and satisfaction with life, according to the participants?

## Methods/Design

### Design

To evaluate the (cost)effectiveness of the life review course 'Looking for Meaning', we carry out a pragmatic randomized controlled trial (RCT) with a cost effectiveness analysis (CEA). Participants are randomly assigned to either the 12-session prevention course or the control condition, the twenty-minute video movie entitled 'The Art of Growing Older'. In addition, we conduct a qualitative interview- and observational study to gain insight into how participants apply and use life review, how they think it helps them coping with depression, and to learn about which course elements have or have not worked for them. The research has been judged and ethically approved by the METiGG, a medical-ethical committee for research in mental health care settings in the Netherlands.

### Participants

Participants are recruited via advertisements in local and national newspapers, magazines targeted at an older audience, or selected with the help of professionals affiliated to one of eleven regional institutes for mental health care in both rural and urban areas of the Netherlands participating in this study. These mental health care institutes support the participant recruitment and -intake, make available the professionals needed, and eventually take care of organizing and offering the course within their institute.

In order to participate in this study, participants have to show slight to moderate depressive symptoms; people diagnosed with a major depression are excluded, as well as people showing no symptoms at all. The intended population is aged 55 and up, as the course was developed for older adults. People who respond, are screened for depressive symptoms by mental healthcare professionals (e.g., a psychologist or a therapist). People scoring 24 or higher on the Center for Epidemiological Studies Depression Scale (CES-D), are further examined with the Mini International Neuropsychiatric Interview (MINI) in order to decide whether a major depression is present or not [19-21]. People with scores below 5 are excluded from the study as well, because improvement is hardly possible. People eligible to participate are asked to complete the consent form.

### Randomization

Participants are randomly assigned to either of the two research conditions by means of a centrally conducted randomization stratified for gender. This means the randomization takes place at the research institute (Netherlands Institute of Mental Health and Addiction), independent of the participating institutions; they receive the outcome of the procedure in the regular mail and are to schedule the participants accordingly.

### Sample size

In order to demonstrate a standardized effect size of 0.35 (a medium-sized effect [22]) a minimum of eighty participants in each condition is required at the time of the follow-up measurement (t2), based on a statistical power (1-beta) of 0.80, a one-sided test, and a conventional alpha of 0.05 (power calculation in Stata 7.0). Anticipating a drop-out of 20% between t0 and t2, slightly over one hundred participants per condition were included at t0, the baseline measurement.

### Blinding

In this study residents, therapists and interviewers collecting data for the qualitative part of the study were not blinded. Therapists are encouraged to present both the experimental and the control condition with equal enthusiasm.

### Experimental condition

Subjects in the experimental condition are assigned to the life review-based prevention course 'Looking for Meaning' for people aged 55 and older [17]. Groups are typically consisting of eight participants. They are conducted by two mental health care professionals with a therapeutic background or an education in behavioral sciences (e.g., psychology) or social work, who for this purpose receive a two-day training program in advance and one day during the course. The 12 sessions of two hours each are similarly structured, including sensory recall exercises, creative activity, and verbal exchange of experiences. Each session is centered on a topic related to the course of life, also making an explicit link between the past and the present.

Meetings are entitled: your name; smells from the past; houses you've lived in; recognizing your resources; hands; photographs; friendship; balance; thread of life and turning points; longing and desire; the future in me; and identity. Participants are encouraged to make use of the senses in order to evoke memories. In addition, creative exercises may open up new ways of expression besides the verbal mode. The course also offers the possibility of further reading and reflection; poems, short stories and literature suggestions are included in the course material.

Based on the experiences of participants in the pilot study [18], three sessions have been replaced or changed. Participants in the pilot indicated that they missed verbal exchange, and would also like to learn about problem solving techniques. These elements were included in the course after the pilot.

### Control condition

The participants assigned to the control group receive an invitation to come watch 'The Art of Growing Older' [23]. This 20-minute educational video movie supplies information about factors and skills that promote growing older successfully. This intervention is considered a minimal intervention, as no treatment is involved.

### Quantitative study

#### Data collection

##### Overview

Table 1 provides an overview of the economic and effect measurements. As a part of the intake and selection interview, we administer the Center for Epidemiological Studies Depression Scale (CES-D), in order to screen the participants. The outcome is considered the CES-D baseline score for those included; the course starts shortly thereafter. Data at t0 (baseline), t1 (effect; 3 months after t0), and t2 (retention; 6 months after t1) are collected by means of a questionnaire. Demographics and additional personal characteristics are recorded at t0 only, and evaluation questions about the participant's experience with the intervention are asked only at t1. Except for that, the three questionnaires are identical.

**Table 1:** Measurement overview per time (intake, t0, t1, and t2) and research section (clinical, economic and qualitative study)

Variables per research section	Instrument	Intake	T0	T1	T2
Screening and selection					
Examine for depression if CES-D ≥ 16	MINI	×			
Clinical effect evaluation					

*Primary outcome*					
Depressive symptoms	CES-D	×		×	×
					
*Secondary outcomes*					
Anxiety	HADS-D		×	×	×
Locus of control; mastery	Pearlin Mastery Scale (5 items)		×	×	×
Satisfaction with life	MANSA (12 items)		×	×	×
					
*Reminiscence functions*	RFS (4/8 subscales)				
Boredom reduction			×	×	×
Bitterness revival			×	×	×
Identity			×	×	×
Problem solving			×	×	×
					
Economic evaluation					

Cost associated with the receipt of care	TIC-P		×	×	×
Quality of life	EQ 5-D		×	×	×
					
Qualitative study					

Interviews with participants			×			
Observation			×			

##### Primary outcome

1. Reduction of depressive symptoms measured using the Center for Epidemiological Studies Depression Scale (CES-D).

The Center for Epidemiologic Studies Depression scale (CES-D) is a 20-item self-report scale developed to measure depressive symptoms in the community [19]. Subjects were asked to indicate how often they experienced each symptom during the previous week. Response categories, ranging form 0 to 3, were 'rarely or never', 'some of the time', 'occasionally', or 'mostly or always'. Summation results in the CES-D score, ranging from 0 to 60. A score of 16 or higher is considered indicative for clinically relevant depressive syndromes. The psychometric properties of the scale are found to be good in older populations [20].

##### Secondary outcome

2. Reduction of anxiety symptoms measured using the Hospital Anxiety and Depression Scale (HADS)

The HADS is a 14-item, self-reporting screening scale comprising two 7-item Likert scales, measuring depression and anxiety, respectively. We use the anxiety scale (HADS-A) [24]. Respondents are asked to indicate whether they experienced feelings of restlessness, tenseness, or panic during the past four weeks. Items range from 0 (rarely or never) to 3 (always or most of the time). The total score ranges from 0 to 21.

3. An increase of satisfaction with life measured using the Manchester Short Assessment of Quality of Life (MANSA).

The Manchester Short Assessment of Quality of Life (MANSA) comprises 16 items: 4 objective questions and 12 subjective questions [25]. We use the 12 subjective items, assessing satisfaction with life as a whole, employment or retirement, financial situation, friendships, leisure, accommodation, personal safety, people that the individual lives with (or living alone), sex life, relationship with family, physical health, and mental health. Each item is rated on a seven-point scale ranging from 1 (couldn't be worse) to 7 (couldn't be better). Summary scores range from 12 to 84; the higher the score the better the quality of life.

4. An increase in perceived control over one's life, measured using the Pearlin and Schooler's Mastery Scale.

The Pearlin and Schooler's Mastery Scale consists of seven items that are intended to assess beliefs of perceived control over one's life in general or beliefs regarding one's ability to control an event [26]. We use the abbreviated version of five items, phrased in a negative way. Possible responses are given on a 5-point scale ranging from 1 (strongly disagree) to 5 (strongly agree). Summary scores range from 5 to 25. Higher scores on the scale indicate lower levels of perceived control.

5. Change or transformation into more positive reminiscence styles measured using the Reminiscence Function Scale (RFS).

The Reminiscence Function Scale is a 43-item questionnaire that can be used to assess reminiscence functions over the life course [27]. The scale comprises eight subscales (factors) reflecting possible functions of reminiscence for the individual. The subscales are labeled 'boredom reduction', 'death preparation', 'identity', 'problem-solving', 'conversation', 'intimacy maintenance', 'bitterness revival', and 'teach/inform'. Questions typically start with "when I reminiscence, it is..." and are completed with 43 possible reasons or motivations to reminiscence. Respondents are asked to indicate the extent to which each of the 43 reasons apply to them. Possible answers range from 1 to 6 (never, rarely, seldom, occasionally, often, or very frequently). For the purpose of this research, 23 questions covering four subscales have been selected: 6 about boredom reduction, 6 about identity, 6 about problem-solving, and 5 about bitterness revival. Examples are: "When I reminisce, it is... to pass the time during idle or restless hours" (boredom reduction), "to see how my past fits in with my journey through life" (identity), "to help me plan for the future" (problem solving), or "to keep painful memories alive" (bitterness revival). Scores are averaged per subscale, representing a reminiscence function each. The higher the score, the more the indicated function applies. In addition, the summary score (all items) indicates the extent to which a person reminiscences irrespective of its reason or function.

##### Additional measures: confounding or modifying variables

In addition to the measurements mentioned above, we assess the following variables that may act as modifying factors of primary and secondary outcomes: Chronic disorders (such as coronary diseases, lung diseases, or rheumatism) critical life events within in the past three years (such as the lost of a spouse, divorce, or having moved), socioeconomic status (SES) according to employment, finances, level of education, and living arrangement.

##### Economic evaluation

The aim of the economic evaluation is to estimate the incremental cost-utility in terms of quality-adjusted life years (Qualy) gained in the treatment group, as compared to the non-treatment group. The economic evaluation is based on the EQ-5d and the Tic-P. Costs of the intervention itself are estimated as well. Measures are described below.

9. An increase of quality of life as measured with the EQ-5D.

The EuroQoL EQ-5D is a pre-scored health classification system that is defined over five domains of health: mobility, self-care, usual activity, pain/discomfort and depression/anxiety [27-29]. Each of the five domains is categorized into three severity levels; 0 (none), 1 (some) and 2 (severe). Responses are combined by a scoring algorithm to produce a summary utility score between -0.594 and 1.0, where 1 represents the state of best imaginable health. Values correspond with Quality-adjusted life years (Qualy) gained or lost. We use an index based on the Dutch population, in accordance with the Dutch TTO tariff [29]. In our study, we also use the EQ-5D as a clinical outcome measure indicating one's perceived quality of life.

10. A decrease in costs of receiving (in)formal health care as measured with the TIC-P.

Cost data are collected using the TIC-P [30]. Costs are defined from a societal perspective and include direct and indirect costs related to receiving formal and informal health care. Production losses in both paid and unpaid work are also covered by the TIC-P. These costs arise when people are absent from work, or work less efficiently due to depression, for example. Production losses will be estimated with the help of the TIC-P.

### Analyses

The analyses will be performed according to the íntention to treat' principle. Therefore, all missing values were imputed. In order to replace the missing values by plausible estimates, we will use the regression imputation procedure as implemented in Stata version 9.1 [31].

We will calculate effect sizes for the immediate improvement of the experimental and the control group on the primary and secondary outcomes by dividing the absolute difference between the post-treatment average score and the pretreatment average score by the pretreatment standard deviation. To establish the maintenance of improvement of both groups, effects sizes will be calculated by dividing the absolute difference between the follow-up average score and the post-treatment average score by the pretreatment standard deviation. For between-group effect sizes, the effect size of the experimental group will be subtracted from the effect size of the control group. Effect sizes of 0.56–1.2 can be assumed to be large, while effect sizes of 0.33–0.55 are moderate, and effect sizes of 0–0.32 are small [32].

In the cost-effectiveness analyses, we will calculate the pre-post changes in costs and identify which respondents show a moderate to large improvement in depressive symptoms in both conditions. These respondents changed reliably, and could thus be considered a 'success'. Then we calculated the incremental cost-effectiveness ratio (ICER) across the experimental and control condition, which represents the incremental costs (or savings) per 'success' (i.e. moderate to large improvement in depressive symptoms) in the experimental condition relative to the control condition. Uncertainty will be assessed by means of non-parametric bootstrapping (2,500 times) of the data of the individual respondents.

### Qualitative study

#### Data collection

To answer research question 4, and gain an insight into the working mechanisms of a life-review approach in general and the course 'Looking for Meaning' in particular, we designed a qualitative study.

The study comprises twenty semi-structured interviews with participants of approximately one hour each, and research observations in three different participant groups during six hours in total. Both the interviews and the observations are centered on participants' experiences with the course, with an emphasis on the life review aspect in relation to the participants' personal experiences and clinical outcome measures. The interview questions are typically open-ended and put in a non-leading way. They leave open *if *life review is helpful or effective at all and gives the participants room to also respond negatively.

The audio-taped interviews are transcribed verbatim. The group observations result in detailed notes about the evaluation process and the interactions between participants. They serve as a point of reference when interpreting the interview data; observations might support or contradict the other qualitative findings.

##### Qualitative data analysis

More than one method may apply to analyze qualitative data, including 'code-and-retrieve' methods (33–35). 'Code and retrieve' means that relevant parts of the material are selected or highlighted (coded), extracted, and clustered in the end. In this process, abductive inference combines new and interesting empirical facts from the data in the first place (bottom-up) with previous theoretical knowledge [34], whereas inductive inference is more restrictive and departs from a theoretical framework (top down) to classify the data.

In this study, we will use a top-down, theory driven approach with an emphasis on newly emerging insight irrespective of whether an observation is made repeatedly or can be easily classified. It is added to the quantitative analysis in order to better understand the outcomes. Relevant information can be recognized in relation to, or in contrast with, the knowledge to date. By reading and re-reading the data materials and making notes with in mind the research questions, the researcher yields descriptive and explanatory research outcomes integrated with his or her theoretical knowledge.

## Discussion

In this paper we described the design of a study on the (cost)effectiveness of a newly developed life-review-based course 'Looking for Meaning'. The results of this study will add to the literature in different ways. Due to the pragmatic character of the Randomized Controlled Trial (RCT), results will show whether potential effects hold in mental health care practice and can be considered ecological valid. We added an economic evaluation, which is the first one regarding life review. In addition, we included a measurement scale based on reminiscence styles, the Reminiscence Function Scale (RFS), to show a change or transformation in reminiscence styles as an outcome of the intervention, which is also a novelty. Finally, we added a qualitative study consisting of interviews with participants and observations of group sessions, because a RCT may show that an intervention is effective, but the reason *why *often remains unclear. The qualitative study enables us to gain more insight into the working mechanisms of the intervention. This insight is important for several reasons. It helps refuting the argument that it was 'mere attention', and not the specific therapeutic approach that has caused the effect. It can also facilitate the selection and motivation of the 'right' participants for the 'right' type of intervention. In addition, it enables scientists and clinicians to improve or adjust the intervention without distorting its effectiveness. The same holds for therapists and other professionals who conduct the course.

Obviously, the research has limitations too. Most of them are related to nonspecific therapeutic benefits that are usually not controlled for in the design of randomized controlled trials, and are inherent in mental health studies of a pragmatic or naturalistic kind. Instead of being put on a waiting list or receiving care-as-usual, for ethical reasons our control group participants are invited to view an informative movie with relevant contents, representing the control condition. In this way, the intervention of which the effectiveness is indicated but not proven yet (Looking for Meaning) is compared with a minimal intervention (movie) of which the effectiveness is unknown. Although a 20-minute movie cannot be expected to rival the impact of a 12-session course, we cannot be sure. Any effectiveness of the movie will lead to an underestimation of the incremental effect size, i.e. the difference between the improvement in the experimental and the control condition. On the other hand, although we encourage therapists to present both interventions with the same enthusiasm, the novelty of the reminiscence course may cause more enthusiasm among the therapists compared to regular courses, leading to an overestimation of the incremental effect size.

The significance of depression for public health especially in older people, underscores the need for preventive interventions. In this study, we investigate the cost(effectiveness) of the indicated prevention course 'Looking for Meaning', which is a well-structured type of life review. Positive results of this study will make an evidence-based intervention to improve public health among older people available.

## Competing interests

The authors declare that they have no competing interests.

## Authors' contributions

AMP drafted the manuscript and supervised the study. A–SM drafted the manuscript and partly analyzed the data of the study. SO helped to draft this manuscript and partly analyzed the data of the study. ETB designed the study and helped to draft this manuscript. All authors read and approved the final manuscript.

## Pre-publication history

The pre-publication history for this paper can be accessed here:


